# The association of job fatigue with mental disorders among bus drivers

**DOI:** 10.5271/sjweh.4065

**Published:** 2022-12-30

**Authors:** Yu-Jen Lin, Tung-Sheng Shih, Wei-Te Wu, Yue-Liang Leon Guo

**Affiliations:** 1 Institute of Environmental and Occupational Health Sciences, College of Public Health, National Taiwan University, Taipei, Taiwan; 2 National Institute of Environmental Health Sciences, National Health Research Institutes, Miaoli, Taiwan; 3 Institute of Labor, Occupational Safety and Health, Ministry of Labor, New Taipei City, Taiwan; 4 Institute of Environmental and Occupational Health Sciences, National Yang Ming University, Taipei, Taiwan; 5 Department of Healthcare Administration, Asia University, Taichung, Taiwan; 6 Environmental and Occupational Medicine, National Taiwan University (NTU) College of Medicine and NTU Hospital, Taipei, Taiwan

**Keywords:** cohort study, job stress, psychiatric disorder, Taiwan

## Abstract

**Objective:**

Mental disorders are a global problem with growing importance. However, the contribution of work factors to the development of mental disorders is inconclusive. This study aimed to assess the impact of fatigue and job stress on mental disorders in a prospective cohort of bus drivers.

**Methods:**

The Taiwan Bus Driver Cohort Study (TBDCS) recruited 1652 bus drivers from a bus company in 2005. Participants self-completed a structured questionnaire in 2007, which included the Demand-Control-Support (DCS) model questions and the Swedish Occupational Fatigue Inventory-Chinese (SOFI-C). Psychiatric care visits and admissions were obtained from the National Health Insurance Research Dataset (NHIRD) from 2003 to 2016 for as a proxy for psychiatric diagnoses. Drivers with a history of psychiatric disorders before the questionnaire survey time were excluded (N=69). During the follow-up period, a new diagnosis with a psychiatric disorder was defined as an event. Cox proportional hazards model was used to estimate the hazard ratio (HR) for new one-set psychiatric disorders, adjusting for age, body mass index, marital status, education, alcohol consumption, smoking, exercise, bus driving experience, shift work, and chronic diseases.

**Results:**

Among the 896 bus drivers retained for analysis, 85 were newly diagnosed with a psychiatric disorders. DCS score was not associated with the risk of developing a psychiatric disorders, but bus drivers with high SOFI-C scores (≥5) had an elevated risk for anxiety or mood disorders (HR 3.35, 95% confidence interval 1.23–9.09).

**Conclusions:**

Among bus drivers, occupational fatigue, as indicated by high a SOFI-C score, might result in an elevated risk of anxiety or mood disorders. Health service organizations should provide recommendations and guidance for drivers with high fatigue levels to avoid anxiety or mood disorders.

In 2016, mental and addictive disorders affected more than 1 billion people worldwide, contributing 7% of the global burden of all diseases and 19% of all disability-adjusted life years (DALY) (1). Depression and anxiety disorders of the DALY were with overall rates of 469 and 271, respectively, per 100 000 male for all people (1, 2). Among transportation workers, the prevalence of depression among men ranged from 2.5% to 13.3% in a systematic review (3). The mental health of transportation workers has potentially higher impacts than other workers, especially bus drivers. Mental illnesses among bus drivers not only affect driving performance but may also pose a danger to the passengers or public property.

Job fatigue and stress are the most commonly mentioned causes for the development of mental illness among professional drivers (4), but information and methodological discrepancies hamper a clear conclusion concerning the contribution of either. There is a large body of research evidence on the association between psychosocial work factors and depression. Among these factors, work stress – defined as a combination of high job demands and low job control – has been found to have a significant effect on depression (5–7). However, past studies have often focused on mixed workplaces after general screening, not on specific workplaces, and lack information about detailed working characteristics and factors for long-term follow-up. These previous studies have pointed out a variety of research limitations, including: (i) a lack of information about work characteristics, (ii) an inability to fully control for previous depression, (iii) measurement of various sources of pressure only in the benchmark year and inability to measure exposure over the entire work period, and (iv) some workers may have left the labor force or changed their jobs (5, 6).

Fatigue is characterized by multidimensional aspects of physical, mental, and functional health, all of which interact with each other (8). In addition, fatigue includes both acute and chronic symptoms, which have subjective and objective characteristics that correspond to individuals (8). Therefore, it is difficult to evaluate the subcomponents of fatigue in a comprehensive assessment. Common job fatigue is the state of feeling very tired, weary or sleepy resulting from insufficient sleep, prolonged mental or physical work, or extended periods of stress or anxiety (9). Extended working hours, a common work-related fatigue or stress factor, has previously been associated with the occurrence of minor psychiatric disorders among truck drivers (10). Although the association of long working hours and depressive disorders or anxiety has been explored, a causal relationship between occupational fatigue and mental disorders has not been fully established. Previous studies have primarily used cross-sectional study designs, which limits their assessment of a causal relationship between physical and mental aspects of fatigue and mental disorders. A longitudinal design is crucial for a causal examination of the possible relationship between fatigue and mental disorders.

This prospective cohort study aimed to assess the effects of job stress and fatigue on the occurrence of mental disorders among bus drivers with a follow-up period of nine years.

## Methods

### Ethics statement

The Institutional Review Board of the National Health Research Institutes in Taiwan approved this study (NIRB File Number: EC1060516-E). The authors confirm that all experiments were carried out in accordance with the relevant guidelines and regulations, including those of the International Conference on Harmonization–Good Clinical Practice. All participants provided informed consent, and respondents were informed of the possible adverse consequences on the interview day. Each individual self-completed a structured questionnaire after providing written informed consent, which included information on age, race, education level, lifestyle habits (smoking, drinking, exercise and drug use), work conditions (first employment and bus driving experience), history of chronic diseases (heart disease, muscle skeleton disease, respiratory disease, kidney disease, liver disease or digestive disease), as well as job stress and fatigue assessments, as detailed below. After completion, the interviewer only conducted a questionnaire item omission check.

### Study population

**Figure 1:**
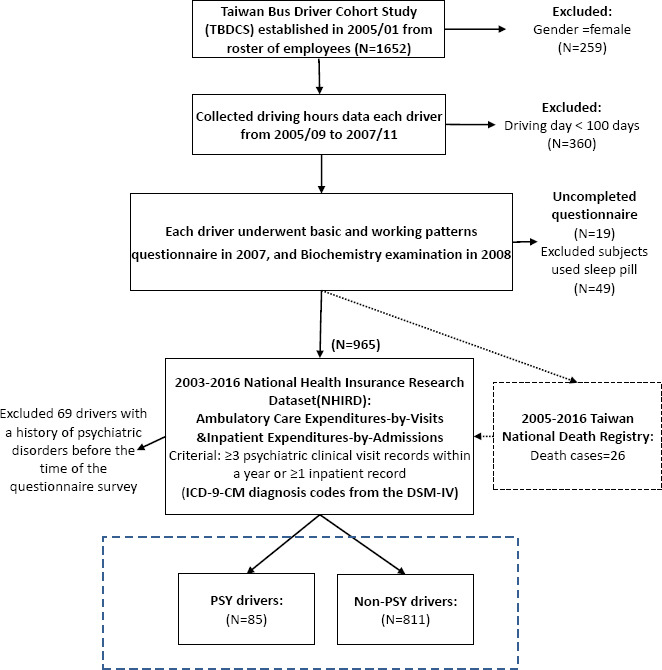
Flowchart of Taiwan Bus Driver Cohort Study (TBDCS). PSY= psychiatric disorders

The Taiwan Bus Driver Cohort Study (TBDCS) recruited 1652 bus drivers from a bus company in 2005. We calculated the driving hours’ dataset of the cohort (total number of records=1 518 350 person-times) based on the Event Data Recorder from 2005 to 2007. The study had four main selection criteria. First, only male bus drivers were selected. Second, the bus drivers must have had >100 driving days during each of the three years. Third, they underwent biochemical tests and completed a self-administered questionnaire. Fourth, they did not use sleeping pills. The subjects self-completed a structured questionnaires in 2007 for job stress using questions from the Demand-Control-Support (DCS) model and for job fatigue using the Swedish Occupational Fatigue Inventory-Chinese (SOFI-C). All subjects were linked to the National Health Insurance Research Dataset (NHIRD), 2003–2016, for psychiatric care visits and admissions as a proxy for psychiatric diagnoses (supplementary material, www.sjweh.fi/article/4065, table S1). Those drivers with a history of psychiatric disorders between 2003 and questionnaire survey time (mostly in 2007) were excluded. Occurrence of new diagnoses of psychiatric disorders was used to define cases (N=85) and controls (N=811) (figure 1).

### Demand-Control-Support model

Participants responded to a series of statements or questions including a work stress assessment. The present study depended on Karasek’s DSC model, which assumes that the conjunction of a high level of psychological demands and a low level of decision freedom leads to psychological stress and stress-related impairments (11, 12). Psychological demands reflect workload, time constraints, and the level of intelligence essentials. Decision latitude is a consolidation of decision authority power and skill discretion. We used the Chinese version of the Job Content Questionnaire (JCQ) which consists of 22 items, including at least one set of questions used to assess the four main JCQ scales: (i) psychological demands (5 items); (ii) job control (9 items); (iii) supervisor support (4 items); and (iv) coworker support (4 items) (13). The Chinese version of JCQ was reliable and valid for assessing psychosocial work conditions among Taiwanese workers with Cronbach’s alpha coefficients >0.80 for all of the JCQ sub-scales in this population, except for the psychological demands subscale (0.55) (13). Psychological demand scores >30 were classified as a high level of job demand and decision latitude scores <60 were classified as a low level of job control; high job stress was defined as having both a high level of job demand and a low level of work control. In contrast, low job stress was defined as either having (i) low job stress (low level of job demand and high level of job control), (ii) active work (high level of job demand and job control), or (iii) passive work (low level of job demand and job control).

### Job fatigue model

Each participant responded to a structured interview that collected a dimensional assessment of the fatigue using the Swedish occupational fatigue inventory (SOFI-C). SOFI-C consists of 25 items in five factors: (i) lack of energy (worn out, exhausted, spent, drained, overworked); (ii) physical exertion (palpitations, sweaty, warm, out of breath, breathing heavily); (iii) physical discomfort (tense muscles, numbness, stiff joints, hurting, aching); (iv) lack of motivation (lack of concern, listless, passive, indifferent, uninterested); (v) sleepiness (lazy, falling asleep, drowsy, yawning, sleepy), with a 11-grade response scale (14). SOFI-C was found to be a useful instrument for measuring fatigue among Chinese sedentary workers with Cronbach’s alpha for the 5-factor scales between 0.88 and 0.95 and the test-retest reliability was satisfactory with intra-class correlations of 0.69–0.83 (15). The two extreme values of the numerical response scale are verbally defined: 0 = “not at all” and 10 = “to a very high degree”. The participants rated the intensity of their perceived fatigue based on 25 items.

### Outcome measures for psychiatric disorders

Psychiatric disorders case data from NHIRD, established by the National Health Insurance Administration, Ministry of Health and Welfare, provides research data for scientists in Taiwan (16). As of 2014, the NHIRD in Taiwan has about 23 million insured persons, covering more than 99% of the Taiwanese population. Two subsets of the database provide key sources for measuring diseases cases, including inpatient by admission and outpatient medical visit by visit. The out- and in-patient-visit datasets are based on the visit form, which includes a record of each admission or visit, along with the ICD-9-CM diagnosis codes from the Diagnostic and Statistical Manual of Mental Disorders IV (DSM-IV). Through each worker’s personal identification number was used to link cases of psychiatric disorders (ICD-9-CM in the first-listed diagnosis code) in the NHIRD between 1 January 2003 and 31 December 2016. Meanwhile, psychiatric disorders events including substance abuse (not including tobacco use disorder) (ICD-9-CM: 291, 292, 303-305), anxiety (ICD-9-CM: 300.00, 300.01, 300.02, 300.21, 300.23, 300.3, 309.81, 308.3, 293.89), mood (ICD-9-CM: 296, 300.4, 301.13, 293.83, 311), anxiety or mood (ICD-9-CM: 300.00, 300.01, 300.02, 300.21, 300.23, 300.3, 309.81, 308.3, 293.89, 296, 300.4, 301.13, 293.83, 311), sleep disorder (ICD-9-CM: 307.42, 307.44, 307.45, 307.46, 307.47, 347, 780.52, 780.54, 780.59) were solely investigated (supplementary table S1). The criteria for psychiatric disorders cases was that study subjects had ≥3 psychiatric clinical visit records within a year or ≥1 inpatient record in the first-listed diagnosis code.

### Statistical analysis

The means and standard deviations were used to describe the distributions of continuous variables. The percentages were used to describe the distributions of categorical variables. Cox proportional hazards model was used to assess the hazard ratio (HR) for psychiatric disorders events among bus drivers with job stress or fatigue, after being adjusted for independent factors. In the Cox model, the follow-up period of the bus driver started from the date of the survey, ie, 1 January 2007 and continued until the date of the first primary diagnosis of psychiatric disease, death or end of follow-up, ie, 31 December 2016 whichever occurred first. The analysis was performed using SAS software (version 9.3; SAS Institute, Cary NC, USA). Occupational psychosocial hazards in theses analyses were high job stress (psychological demands >30 and decision latitude <60) and high fatigue (SOFI-C ≥5). Four models were analyzed: (i) model 1 – high job stress or high fatigue were investigated for psychiatric disease incidences adjusted for age, body mass index (BMI), marital status, and education; (ii) model 2 – model 1 and additionally adjusted for drinking, smoking, and exercise; (iii) model 3 – model 2 and additionally adjusted for bus driving experience, shift work, and history of chronic diseases.

## Results

The demographic characteristics, including age, marital status, education level, BMI, work duration, and work patterns, were similar between drivers with and without psychiatric disorders (table 1). Table 2 shows the job stress and fatigue scores. For the job stress of DCS model, no differences were found between drivers with and without psychiatric disorders. In job fatigue of SOFI-C, the two groups did not differ in continuous or category score, but 43.5% of drivers with psychiatric disorders and 35.5% drivers without psychiatric disorders had a high SOFI-C score (≥5).

**Table 1:** Baseline characteristics and biochemical indices among bus drivers. [PSY=psychiatric disorders]

Variables	All drivers (N=965)		Non-PSY drivers (N=811)		PSY drivers (N=85)		P-value
		
	N	%	N	%	N	%	
Age (years)							
<35	195	20.21	165	20.35	16	18.82	0.915
35–44	446	46.22	370	45.62	38	44.71	
45–49	202	20.93	174	21.45	21	24.71	
≥50	122	12.64	102	12.58	10	11.76	
Body mass index (kg/m^2^)							
<25	397	41.14	336	41.43	34	40.00	0.138
25–29.9	420	43.52	358	44.14	32	37.65	
≥30	148	15.34	117	14.43	19	22.35	
Marital status							
Unmarried	174	18.03	150	18.50	11	12.94	0.432
Married	683	70.78	570	70.28	63	74.12	
Others	108	11.19	91	11.22	11	12.94	
Education							
≤ Junior high school	263	27.25	217	26.76	26	30.59	0.541
Senior high and vocational school	629	65.18	534	65.84	51	60.00	
University and College	73	7.56	60	7.40	8	9.41	
Cigarette smoking							
Never smokers	330	34.20	273	33.66	33	38.82	0.705
Ex-smokers	67	6.94	57	7.03	6	7.06	
Current smokers	562	58.24	476	58.69	46	54.12	
Alcohol use							
No	739	76.58	620	76.45	63	74.12	0.547
Current	219	22.69	185	22.81	22	25.88	
Moderate exercise							
No	687	71.19	591	72.87	54	63.53	0.063
Yes	266	27.56	210	25.89	30	35.29	
Age at first employment (years)							
≤32	242	25.08	207	25.52	17	20.00	0.531
33–38	325	33.68	271	33.42	30	35.29	
≥39	398	41.24	333	41.06	38	44.71	
Time since first employment (years)							
≤2	216	22.38	181	22.32	17	20.00	0.258
2.1–5	289	29.95	249	30.70	21	24.71	
5.1–8	188	19.48	150	18.50	23	27.06	
>8	272	28.19	231	28.48	24	28.24	
Shift work mode							
Day shift only	114	11.81	97	11.96	8	9.41	0.457
Irregular shift	793	82.18	664	81.87	73	85.88	
History of chronic disease							
No	700	72.54	585	72.13	64	75.29	0.535
Yes	265	27.46	226	27.87	21	24.71	

**Table 2:** Baseline of job demand, job resources and fatigue score between drivers with psychiatric disorders (PSY) and non-PSY drivers.

	Non-PSY drivers (N=811)				PSY drivers (N=85)				P-value
	
	Mean	SD	N	%	Mean	SD	N	%	
Demand-Control-Support Model									
Job Demand									
Psychological demands	30.10	3.30			30.47	3.62			0.326
Job Control									
Decision latitude	59.00	8.02			59.43	9.24			0.646
Job Support									
Supervisor support	11.33	1.56			11.31	1.60			0.885
Co-worker support	10.44	1.44			10.45	1.27			0.933
Job Fatigue Model									
SOFI	3.60	6.33			3.47	2.71			0.730
Demand-Control-Support Model									
Psychological demands									
≤30			502	61.90			51	60.00	0.732
>30			309	38.10			34	40.00	
Decision latitude									
≤60			488	60.17			45	52.94	0.200
>60			315	38.84			39	45.88	
Job strain									
Low strain			607	74.85			63	74.12	0.905
High strain			196	24.17			21	24.71	
Job Fatigue Model									
SOFI									
<3			355	43.77			35	41.18	0.272
3–4.9			168	20.72			13	15.29	
≥5			288	35.51			37	43.53	

### Associations of DCS and SOFI-C variables with psychiatric disorders

Table 3 shows the association of DCS and SOFI-C model variables with psychiatric disorders, adjusted for age, BMI, education, alcohol consumption, smoking, exercise, length of first employment, shift work, and history of chronic diseases. In the fully adjusted model, SOFI ≥5 was associated to non-significant increase of the incidence of psychiatric disorders (HR 1.52, 95% CI 0.93–2.49).

**Table 3:** Hazard ratio (HR) and 95% confidence interval (CI) for psychiatric disorder by job stress in bus drivers.

Independent variable ^a^	Model 1 ^a^		Model 2 ^b^		Model 3 ^c^	
		
	HR	95% CI	HR	95% CI	HR	95% CI
Demand-Control-Support Model						
Psychological demands >30 (reference≤30)	1.06	0.68–1.65	1.07	0.69–1.67	1.15	0.73–1.81
Decision latitude >60 (reference ≤60)	1.34	0.87–2.06	1.29	0.83–2.01	1.31	0.83–2.06
High job strain (reference = low strain)	1.01	0.61–1.66	1.03	0.62–1.69	1.12	0.64–1.70
Job Fatigue Model						
SOFI (reference <3)						
3–4.9	0.83	0.44–1.58	0.84	0.44–1.60	0.98	0.51–1.90
≥5	1.38	0.85–2.21	1.40	0.86–2.26	1.52	0.93–2.49

a Each independent variable (1–4) was solely included in the models.

b Adjusted for age, body mass index, marital status, and education.

c Model 1 and additionally adjusted for drinking, smoking, and exercise.

^d^ Model 2 and additionally adjusted for bus driving experience, shift work and history of chronic diseases.

### Associations of DCS and SOFI-C variables with anxiety or mood disorders

Table 4 showed the relationship of DCS and SOFI-C model variables with anxiety or mood disorders, adjusted for age, BMI, education, drinking, smoking, exercise, time since first employment, shift work, and history of chronic diseases. A high SOFI-C score (≥5) was closely related with an increased risk of anxiety or mood disorders among bus drivers, even after controlling for potential confounders (model 3: HR 3.35, 95% CI 1.23–9.09).

**Table 4:** Hazard ratio (HR) and 95% confidence interval (CI) for anxiety or mood disorders by job stress in bus drivers.

Independent variable ^a^	Model 1 ^a^		Model 2 ^b^		Model 3 ^c^	
		
	HR	95% CI	HR	95% CI	HR	95% CI
Demand-Control-Support Model						
Psychological demands >30 (reference≤30)	1.39	0.62–3.14	1.47	0.65–3.32	1.63	0.71–3.72
Decision latitude >60 (reference ≤60)	1.61	0.72–3.60	1.78	0.78–4.04	1.95	0.85–4.43
High job strain (reference = low strain)	1.24	0.51–3.00	1.22	0.50–2.96	1.38	0.56–3.37
Job Fatigue Model						
SOFI (reference <3)						
3–4.9	1.85	0.56–6.08	1.82	0.55–6.00	2.15	0.64–7.22
≥5	2.96	1.10–7.95	2.93	1.08–7.92	3.35	1.23–9.09

a Each independent variable (1–4) was solely included in the models.

b Adjusted for age, body mass index, marital status, and education.

c Model 1 and additionally adjusted for drinking, smoking, and exercise.

^d^ Model 2 and additionally adjusted for bus driving experience, shift work and history of chronic diseases.

## Discussion

This prospective study found that job fatigue is a significant positive predictor of the development of anxiety or mood disorders among bus drivers during 9-year long-term follow-up. Higher risk of developing anxiety or depressive disorders was found among those with high fatigue (SOFI-C ≥5) as compared with those with low fatigue. SOFI-C is a comprehensive instrument for evaluating subjective perceptions of fatigue in the workplace, where multiple environmental factors interplay. It can be used to quantify instant status or short-termed fatigue symptoms rather than relevant causes or consequences and has been demonstrated to prevent occupational injuries caused by fatigue among a population of Asian workers in the workplace (15). Job fatigue is a form of physical and mental burnout exacerbated by workplace conditions, work stress, high workloads, long working hours, and chronic illness, with the nature of fatigue varying between workplace. For long-haul drivers, the nature of the job is long-distance driving and sedentary work, requiring long hours of vigilance and mental control, which mainly leads to mental fatigue (17). Unlike physically demanding occupations such as firefighters and heavy manual labor, there is a significant high correlation with physical fatigue (18, 19).

Using the NHIRD database, it was possible to estimate a prevalence rate for psychiatric disorders of 4.3% among males ≥18 years in 2000 in Taiwan’s general population. For this, we used the definition of ≥1 service for ambulatory or inpatient care with a principal diagnosis of a psychiatric disorder, including ICD-9-CM codes 290–298; minor psychiatric disorders under our criteria included ICD-9-CM codes 300–316, excluding 303–305 and 312–315 (20). The present study showed the prevalence of mental disorders to be 8.8% among bus drivers from 2007 to 2016, more than double than that of the general population.

Job fatigue among bus drivers can be attributed to a several factors including long working hours, lack of sleep or poor sleep (21–23). A longitudinal study of 2960 full-time employees with a 5-year follow-up found that working long hours is a risk factor for developing of anxiety symptoms among men (24). A prospective Whitehall II cohort study with a baseline examination of working hours and the follow-up of major depressive episodes in 1997–1999 (mean follow-up 5.8 years) found over a double increased risk of major depressive episode for those working >11 hours a day compared to employees working 7–8 hours a day, which remained robust after adjustment for chronic physical disease, smoking, alcohol use, job stress, and work-related social support had little effect on this association (OR 2.52, CI 1.12–5.65) (25). Among Norwegian working men, a cross-sectional study using the Hospital Anxiety and Depression Scale (HADS) found that those working 49– 100 hours per week as compared with those working 35-40 hours per week had a significantly higher prevalence of anxiety (prevalence 13.4% versus 20.6%, OR 1.67, CI 1.36–2.06) and depressive disorders (prevalence 9.1% versus 13.1%, OR 1.50, CI 1.17–1.93) (26). The present study showed similar results associated with the long working hours (driving time of >10 hours per day: 43.8% of every day in high fatigue (SOFI-C ≥5) (supplementary table S2).

Our study did not find an association between job stress and subsequent psychiatric disorders in Taiwan bus drivers. In contrast, a previous longitudinal epidemiologic study found an association between work-related psychological stress factors and the development of depression (27). This is most likely due to the low variability of working conditions and high remuneration of bus drivers in Taiwan. We suspect that social class and Eastern culture may confound the association between work stress and mental illness, and that even these factors may be so highly correlated that it is difficult to distinguish their independent effects in this study.

Previous research views fatigue and mood dysregulations as co-associated disorders that represent clinical manifestations of shared neurological pathologies related to inflammation, oxidative stress, and abnormal activity of the hypothalamic–pituitary–adrenal axis (28). Among them, inflammation has been hypothesized as a possible contributor to fatigue (29–31). Different mechanisms have been proposed, including inflammatory factors acting on the central nervous system (CNS) (32–34). Cytokine imbalance affects not only inflammation but also dopamine and serotonin production (35, 36). Dopamine and serotonin are two neurotransmitters that affect similar aspects of human health in slightly different ways, including your mental health, digestion, and sleep cycle (37, 38).

This investigation has several strengths. Firstly, a prospective cohort study design was used to collect information on different dimensions of bus drivers’ working environment, including work stress (JCQ) and work fatigue (SOFI-C), to provide a clear causal relationship between exposure and disease. SOFI-C is a valuable instrument for measuring fatigue among workers (15). The 5-factor structure of SOFI-C correlates strongly with changes in fatigue-related physiological parameters, including electroencephalogram and electromyography measures (39). Secondly, using NHIRD diagnoses by psychiatrists from a long-term follow-up approach provided medical assessment independent of the work exposure measured earlier. Thirdly, the coverage of NHIRD registry data has been nearly complete, including 99% of the population (40). Our study participants are all covered by the national health insurance. Therefore, the concerns of loss-to-follow-up are minimized.

In addition, we fully admit that there are limitations to this study. First, the measurement of fatigue by using SOFI-C was carried out in one period and might not have been representative of longer-term fatigue among the participants. A previous study indicated that the symptoms of fatigue could persist beyond five years from the initial time of encounter (4, 41). Second, this investigation only recruited male drivers restricting the results’ generalization to females. Third, the small number of cases in these sub-categories might be a statistical power restriction. This might have limited the observed relationship between all psychiatric disorders and job stress and fatigue. Nevertheless, a significant association was found between fatigue and anxiety or mood disorders. Fourth, it is a concern that loose criteria of psychiatric diagnoses could have caused false inclusion of cases. Therefore, we used the strict criteria that psychiatric disorders cases had to have ≥3 visits for the same diagnosis within one year or were an inpatient with ≥1 admissions during the study period based on a clinical physician’s suggestion. This increased the accuracy of case inclusion, but it could have caused underestimation of the effects. It can also detect occupational injuries caused by workers’ fatigue in the workplace, but less attention has been paid to long-term occupational diseases. This study found that SOFI-C was predictive of subsequent development of psychiatric disorders. Therefore, these findings provide evidence that driver-fatigue countermeasures can be applied through drivers, transport companies, and government programs to reduce mental disorders, such as through publicity campaigns, road-infrastructure measures, vehicle-based detection and warning devices, legislation, and enforcement.

### Concluding remarks

This study revealed that work-related fatigue and job stress were prevalent among bus drivers in Taiwan. A significant relationship was found between anxiety or mood disorders with fatigue but not job stress. These findings emphasize the need for interventions aimed at reducing fatigue as it has the potential to lead to mental disorders in this working population.

## Supplementary material

Supplementary material
