# Supplementary material for: The association of job fatigue with mental disorders among bus drivers
Source: Scand J Work Environ Health. 2022 Dec 30;49(1):75–83. doi: 10.5271/sjweh.4065 (PMC10549917; doi:10.5271/sjweh.4065)
Supplement: Supplementary material [file SJWEH-49-75-S001.pdf]

The association of job fatigue with mental disorders among bus drivers[1]

by Yu-Jen Lin, MSc, Tung-Sheng Shih, PhD, Wei-Te Wu, PhD, Yue-Liang Leon Guo, MD, PhD[2]

1. Supplementary material

2. Correspondence to: Yue-Liang Leon Guo, Environmental and Occupational Medicine, National Taiwan University (NTU) College of Medicine and NTU Hospital, Rm 339, 3F., No. 17, Xuzhou Rd., Zhongzheng Dist., Taipei City 10005, Taiwan. [E-mail: leonguo@ntu.edu.tw]

Table S1 Cases of psychiatric disorders disease in bus driver from 2007 to 2016 (n=896) [#]

	**ICD-9**	**Case** [a.]
**Total**		**85**
(1)Substance abuse	291, 292, 303, 304, 305 (Not included 305.1)	*
(2)Anxiety	300.00, 300.01, 300.02, 300.21, 300.23, 300.3, 309.81, 308.3, 293.89	20
(3)Mood	296, 300.4, 301.13, 293.83, 311	9
(2)Anxiety or (3)Mood	300.00, 300.01, 300.02, 300.21, 300.23, 300.3, 309.81, 308.3, 293.89<br>296, 300.4, 301.13, 293.83, 311	24
(4)Sleep disorder	307.42, 307.44, 307.45, 307.46, 307.47, 347, 780.52, 780.54, 780,59	68

[a.] The current analysis was based on National Health Insurance Database provided by the Collaboration Center of Health Information Application, Ministry of Health and Welfare, Executive Yuan, Taiwan, the candidates must have at least three clinical visit records within a year.

* According to the government regulation, it cannot be labeled as less than 2.

[#] Excluded 69 drivers with a history of psychiatric disorders

Table S2 The distribution of driving hours a day in bus driver across SOFI scales (n=965)

SOFI-C	<3		3-4		>=5		
	N	(%)	N	(%)	N	(%)	p-value
Driving more than 10 hours a day							<0.001
Never	26	6.22	9	4.66	3	0.85	
Seldom（1-3 times/months）	51	12.20	16	8.29	13	3.69	
Occasionally（1-2 times/weeks）	97	23.21	45	23.32	42	11.93	
Regularly（3-5 times/weeks）	160	38.28	79	40.93	140	39.77	
Always（Each day）	84	20.10	44	22.80	154	43.75	
